# The Persian version of the centrality of event scale (CES): Assessment of validity and reliability among Iranian university students

**DOI:** 10.1002/brb3.2448

**Published:** 2021-11-25

**Authors:** Zahra Azadfar, Zohreh Khosravi, Azam Farah Bijari, Abbas Abdollahi

**Affiliations:** ^1^ Department of Psychology Faculty of Education and Psychology Alzahra University Tehran Iran; ^2^ Department of Counseling Faculty of Education and Psychology Alzahra University Tehran Iran

**Keywords:** centrality of event scale, Iranian, psychometrics, reliability, university students, validity

## Abstract

**Background:**

Event centrality, the extent to which an experience is perceived as a central event in one's life, has been found to be a strong correlate of posttraumatic stress disorder (PTSD). The centrality of event scale (CES) is commonly used in different conditions and cultures to measure trauma‐related effects. However, the psychometric properties of this scale have not been investigated in the Iranian context.

**Methods:**

The present study examined the psychometric properties of the Persian translation of the 7‐item CES in a sample of 525 university students with a history of a romantic breakup.

**Results:**

Confirmatory factor analysis confirmed a one‐factor structure. The CES score was positively correlated with psychological inflexibility and PTSD symptoms. The measurement invariance analyses showed that the 7‐item CES is gender invariant and can be used for both men and women.

**Findings:**

Findings supported the good psychometric properties of the 7‐item CES for measuring event centrality in Iranian university students.

## Abstract 2

The present study examined the psychometric properties of the Persian translation of the 7‐item CES in a sample of 525 university students with a history of a romantic breakup.

## 1 INTRODUCTION

The prominent theoretical models of PTSD have emphasized the key role of cognitive factors and traumatic memory in the development and maintenance of PTSD symptoms, to the extent that PTSD has been described as a disorder related to autobiographical memory (Brewin, 2007; Rubin et al., 2008). According to many traditional PTSD theories, a traumatic event may violate an individual's schemas, worldview, and self‐knowledge, and therefore may be poorly integrated into autobiographical memory, resulting in the formation of fragmented memories and incoherent life stories (Janoff‐Bulman, 1989; Van der Kolk & Fisler, 1995; Ehlers & Clark, 2000; for a review, see Dalgleish, 2004). Alternatively, Berntsen and Rubin (2006) proposed a different viewpoint by introducing the construct of *event centrality*. They claimed that traumatic memory does not necessarily lead to poor integration, but forms highly accessible vivid memories due to their emotionality and distinctiveness. Such memories organize a reference point for attributing meaning to other life experiences, served as a turning point in the life story, and become a central component of personal identity (Berntsen & Rubin, 2006, 2007).

Event centrality has been evidenced as one of the significant correlates of post‐traumatic stress symptoms (PTSS) in populations with different traumatic events, including survivors of childhood maltreatment (Watts et al., 2021), individuals diagnosed with PTSD (Uzer et al., 2020), survivors of natural disasters (Mordeno et al., 2018), refugees (Chung & Shakra, 2020), and bereaved undergraduates (Bellet et al., 2018). Research has shown that event centrality positively correlates with PTSD symptoms, even when controlling for event severity, depression, anxiety, dissociation, self‐consciousness, and personality traits (Berntse & Rubin, 2007; Ogle et al., 2013; Vermeulen et al., 2020). A review has found that the centrality of past negative events is associated with a variety of post‐trauma psychopathologies, including PTSD, depression, anxiety, and complicated grief (Gehrt et al., 2018). Moreover, the predictive role of CES in the development of PTSD symptoms has been demonstrated in some prospective studies (Blix et al., 2016; Boals, 2014; Boals & Ruggero, 2016; Boelen, 2012). Few studies have also established the causal role of event centrality in the development and maintenance of PTSD symptoms (Boals & Murrell, 2016).

Several measures are currently used in Iran to assess exposure to traumatic events and the impact of such experiences, including the impact of event scale‐revised (IES‐R; Panaghi et al., 2006), and the posttraumatic stress disorder checklist for DSM‐5 (PCL‐5; Varmaghani et al., 2018). However, assessment of trauma exposure alone cannot capture the influence of personal perception and evaluation of the event on post‐trauma psychopathologies. According to event centrality theory, exposure to potentially traumatic life events does not necessarily lead to the development of PTSD, rather the objective stressor should be subjectively appraised as an event with high perceived importance (Berntsen & Rubin, 2006; Boals, 2018; Creamer et al., 2005). The centrality of event scale (CES) is the only measure consistent with the theoretical framework of event centrality that assesses three aspects of traumatic memories (Berntsen & Rubin, 2006).

The original English version of the 7‐item CES was first developed by Berntsen and Rubin (2006) and validated on 707 undergraduates from four North American universities. The results of the exploratory factor analysis (EFA) indicated a unifactorial structure with good psychometric properties. The internal consistency coefficient of this measure was 0.88. Furthermore, the 7‐item CES was highly correlated with the full 20‐item version (*r *= 0.96). The results of the concurrent validity demonstrated that the 7‐item CES was positively correlated with PTSD and depressive symptoms. Additionally, individuals who scored above the cutoff point of PTSD and individuals who reported experiencing events corresponding to the A2 criterion (involving intense fear, helplessness, or horror) had higher CES scores (Berntsen & Rubin, 2006).

The psychometric properties of the 7‐item CES have been supported in several studies among English‐speaking and non‐English‐speaking university students and adolescents (Berntsen & Rubin, 2006; Galán et al., 2017; Gauer et al., 2013; Vagos et al., 2018; Vermeulen et al., 2020), and these studies reported good to excellent internal consistency for the 7‐item CES. The Cronbach's alpha value was 0.88 in the English version (Berntsen & Rubin, 2006), 0.89 in the Brazilian Portuguese version (Gauer et al., 2013), 0.84 in the Spanish version (Galán et al., 2017), 0.85 to 0.93 in the Dutch version (Vermeulen et al., 2020), and 0.90 in the Portuguese adolescents’ version (Vagos et al., 2018).

The main purpose of the current study was to measure the psychometric properties of the Persian version of the 7‐item CES among Iranian university students with a history of at least one romantic breakup. The prevalence of PTSD symptoms is higher among individuals who have experienced an interpersonal stressful life event compared to non‐interpersonal experiences (Ogle et al., 2013). One reason is that interpersonal events are more likely to be considered as a central event in personal identity, especially among young adults for whom establishing and maintaining interpersonal relationships is a developmental task (Reiland & Clark, 2017). Romantic breakup is one of the most common interpersonal stressful life events among university students (Anders et al., 2012). A review found that two‐thirds of university students have experienced a romantic breakup in the past three months (Field et al., 2009). Moreover, Chung et al. (2002) found that more than 70% of people experience high levels of PTSS after a romantic breakup. Although romantic breakup is not a life‐threatening event and may not meet the A criteria for trauma in the DSM‐5 (American Psychiatric Association, 2013), recent research has documented that low magnitude life events, such as romantic breakup, can result in comparable even more severe symptoms than criterion A events (Gold et al., 2005; Long et al., 2008; Spitzer et al., 2000). Anders et al. (2011) found that people often consider relationship conflict or romantic breakup as the worst event they have ever experienced. Moreover, Berntsen and Rubin (2006) showed that the level of CES did not depend on whether or not respondents reported having experienced an event that met the A1 criterion of the DSM‐IV (actual or threatened death or serious injury). Indeed, event centrality has provided a potential clarification of the dispute over criterion A (Berntsen & Rubin, 2006; Boals, 2010). Researchers have reported that event centrality also positively correlates with PTSD symptoms in the context of non‐traumatic life events such as the loss of a loved one (Boelen, 2009) and persistent pain (Perri & Keefe, 2008). In addition, Boals (2014) found that event centrality increased the risk of PTSD and depressive symptoms following relationship conflict or dissolution in a sample of 312 nonclinical volunteers. Therefore, we validated the 7‐item CES in a sample of Iranian university students who had experienced at least one romantic breakup in the past 2 years.

Validation of a measure for assessing event centrality in individuals who have experienced a stressful or traumatic life event may contribute to understanding the underlying mechanisms in the development of posttraumatic psychopathologies. However, there is currently no Persian measure to assess the role of traumatic memories in the development of posttraumatic symptoms. Existing psychometric studies have been conducted in Western cultures. Assessing the validity and reliability of the 7‐item CES in an Eastern culture may help determine its consistency across cultures and languages. In addition, the short version of the CES may be easy to administer and reduce the burden of assessment. Therefore, the present study sought to assess the psychometric properties of the Persian version of the 7‐item CES among Iranian university students with a romantic breakup. We hypothesized that the 7‐item CES would have a one‐factor structure and adequate internal consistency in this population (Hypothesis 1).

One important factor that is related to both event centrality and developing PTSD symptoms following stressful experiences is psychological inflexibility (Boykin et al., 2019; Schramm et al., 2020). Psychological inflexibility is composed of six interrelated processes: experiential avoidance, lack of contact with the present moment, self as content, cognitive fusion, inaction, and lack of contact with values (Hayes et al., 2012). Experiential avoidance is related to the avoidance of trauma‐related thoughts, feelings, and memories, which increase the risk for PTSD symptoms following a stressful event (Schramm et al., 2020). Avoidance of external trauma‐related reminders (people, places, and situations) and internal stimuli (associated memories, thoughts, and feelings) is an important diagnostic feature of PTSD (American Psychiatric Association, 2013). Several studies support the positive association between psychological inflexibility and PTSD symptoms (Bryan et al., 2015; Thompson & Waltz, 2010; Walser & Westrup, 2007). A preliminary longitudinal study found that reducing psychological inflexibility may contribute to improving PTSD symptoms over the course of treatment (Schramm et al., 2020). Psychological inflexibility has been shown to increase the risk of developing PTSD symptoms following experiences with high centrality (Boykin et al., 2019). The centralization of a traumatic experience in self‐identity is a key aspect of event centrality (Berntsen & Rubin, 2006, 2007). Similarly, attachment to a self‐conceptualization as a process of psychological inflexibility reflects defining the self‐identity based on an experienced event. Accordingly, over‐identification with an experience increases the risk for developing posttraumatic symptomatology (Boykin et al., 2019). Boals and Murrell (2016) demonstrated that a brief acceptance and commitment therapy (ACT) with particular focus on the self as context led to improvement in PTSD and depressive symptoms by reducing event centrality. Therefore, we hypothesized that CES scores would be positively correlated with psychological inflexibility and PTSD symptoms (Hypothesis 2).

While most studies have found nonsignificant or small gender differences in event centrality (Berntsen et al., 2011; Cunha et al., 2015; Gauer et al., 2013; Vagos et al., 2018; for a review, see Gehrt et al., 2018), some research has shown that women are more likely than men to view a negative event as central to their identity (Boals, 2010). Therefore, it seems essential to further investigate gender differences in the centrality of negative events between men and women. We hypothesized that the 7‐item CES would be gender invariant and that there would be no significant differences between male and female CES scores (Hypothesis 3).

## 2 METHOD

### 2.1 Participants

Participants included 525 university students (400 women) aged 18 to 25 years (M = 21.91, SD = 2.27) from several universities in Tehran who had experienced at least one nonmarital relationship breakup in the past two years. The demographic characteristics of the participants are presented in Table 1.

**TABLE 1:** Participant demographics

Variable	Percent (*n*)
**Relationship duration**	
Less than 6 months	30% (*n* = 157)
Between 6 months to 1 year	21% (*n* = 108)
Between 1 year to 2 years	19% (*n* = 100)
More than 2 years	30% (*n* = 160)
**Relationship commitment and intimacy**	
Low	13% (*n* = 72)
Moderate	37% (*n* = 193)
High	50% (*n* = 260)
**The initiator status**	
Initiator	51% (*n* = 265)
Non‐initiator	49% (*n* = 260)
**Breakup distress**	
Low	7% (*n* = 39)
Moderate	24% (*n* = 124)
High	69% (*n* = 362)

### 2.2 Instruments

#### 2.2.1 The centrality of event scale‐short version

(CES; Berntsen & Rubin, 2006). This measure consists of seven self‐reported items (e.g., “*I feel that this event has become part of my identity*”) based on a 5‐point Likert scale ranging from 1 (“*Totally disagree*”) to 5 (“*Totally agree*”). CES measures the extent to which the memory of a stressful or traumatic life event becomes a central point of one's life story and identity and forms a reference point for organizing autobiographical knowledge. A higher score on this scale represents a higher degree of event centrality. In the original study, an internal consistency of 0.88 was reported for the 7‐item version (Berntsen & Rubin, 2006).

The Brislin (1986) method of translation was administered to translate the English version of the CES into Persian. Two professional translators who were experts in both English and Persian languages independently translated the CES. One of them translated this measure from English to Persian and the other translator, unaware of the first translation version, back‐translated the Persian version to English. Eventually, three independent translators compared these two versions and reported no significant differences between the original scale and the Persian one in terms of content and concept.

#### 2.2.2 The posttraumatic stress disorder checklist for DSM‐5

(PCL‐5; Blevins et al., 2015; Weathers et al., 2013). This measure consists of 20 items (e.g., “*Repeated, disturbing, and unwanted memories of the stressful experience?*”) that use a 5‐point Likert scale ranging from 0 (“*Not at all*”) to 4 (“*Extremely*”) to assess the severity of PTSD symptoms. This scale, based on the DSM‐5 model, covers four subscales to assess criteria B through E, including re‐experiencing, avoidance, negative alterations in cognition and mood, and hyperarousal. A higher score on this scale indicates a higher severity of PTSD symptoms (Blevins et al., 2015; Weathers et al., 2013). An Iranian version of this scale was used with internal consistency coefficients ranging from 0.67 to 0.90 for the subscales (Varmaghani et al., 2018). In the present study, the Cronbach's alpha coefficients were 0.85 (re‐experiencing), 0.83 (avoidance), 0.90 (negative alterations in cognitions and mood), 0.80 (hyperarousal), and 0.93 for total PTSD.

#### 2.2.3 The acceptance and action questionnaire

(AAQ‐II; Bond et al., 2011). This measure consists of seven items (e.g., “*My painful memories prevent me from having a fulfilling life*.”) that are rated on a 7‐point Likert scale ranging from 1 (“*Never true*”) to 7 (“*Always true*”). A lower score indicates more psychological flexibility and a higher score indicates more psychological inflexibility (Bond et al., 2011). An Iranian version of this scale with a Cronbach's alpha of 0.86 was used in this study (Imani, 2016). In the present study, the Cronbach's alpha coefficient for this scale was 0.92.

### 2.3 Ethical considerations

The procedure and research materials of the present study were reviewed and approved by the Ethics Committee at Alzahra University. Participants were assured that participation in this study was voluntary and that their responses would be kept anonymous and confidential.

### 2.4 Procedure

A community sample of university students aged 18 to 25 who had experienced at least one romantic breakup in the past two years was invited to participate in a study about the romantic breakup. The online survey was conducted on the online survey platform Porsa. An advertisement that included a brief description of the purpose of the research and a link to the online questionnaires was shared on the social networks of several universities in Tehran. Participants were instructed to reflect upon their recent romantic breakup while completing the CES and PCL‐5 questionnaires. The average time taken by university students to complete the questionnaires was 20 min. As compensation, participants had the opportunity to enter a drawing for gift cards. Data collection was conducted from April to May 2021.

## 3 RESULTS

### 3.1 Face validity

The face validity of the translated 7‐item CES was decided using two qualitative and quantitative methods. In the qualitative part, eight university students were interviewed about the comprehensibility and difficulty of each item. Minor changes were made based on the students’ views. In the quantitative part, the same eight university students were asked to rate the importance of the items (difficulty, relevance, appropriateness, and comprehensibility) on a 5‐point Likert scale ranging from 1 (not important) to 5 (completely important). The impact score index was calculated using the following formula: Impact score = frequency (%) × importance. In this formula, frequency represents the number of participants who chose a value of 4 or 5 for an item, and importance represents the mean value of that item. According to Hajizadeh & Asghari (2011), a value of 1.5 or more for each item indicates acceptable face validity for that item. The results revealed that all indicators of the translated 7‐item CES had an impact score of more than 1.5, which indicates adequate face validity.

### 3.2 Content validity

The content validity of the translated 7‐item CES was determined using both qualitative and quantitative methods. In the qualitative part, eight experts (psychologists) were asked to examine the measure and provide comments on grammar, word usage, simplicity, and clarity of each item. After applying the aforementioned corrections from the experts’ perspectives, a questionnaire was created to determine quantitative content validity. For this purpose, the content validity index (CVI) and the content validity ratio (CVR) were used. To estimate the CVI, the same eight experts were asked to rate the simplicity, clarity, and relevance of the indicators on a 4‐point Likert scale ranging from 1 (not relevant at all) to 4 (highly relevant). The formula for assessing CVI is as follows: The total number of experts who gave a score of 3 or 4 is divided by the total number of experts. As shown in Table 2, the CVI scores for all indicators were greater than 0.7 (Polit et al., 2007), indicating acceptable content validity for all items. The CVR estimates the essentiality of items, which were rated by the eight psychologists on a 3‐point scale ranging from 1 (not essential) to 3 (essential). The value of CVR was calculated using the following formula:

$$\mathrm{CVR}=\frac{ne-N/2}{N/2}$$

**TABLE 2:** CVR and CVI for the items of centrality of events scale (CES)

		CVI^a^			CVR^b^
No	Items	Simplicity (1–4)	Relevancy (1–4)	Clarity (1–4)	Essential (1–3)
1	I feel that this event has become part of my identity.	1.00	1.00	1.00	1.00
2	This event has become a reference point for the way I understand myself and the world.	1.00	1.00	1.00	1.00
3	I feel that this event has become a central part of my life story.	1.00	1.00	1.00	1.00
4	This event has colored the way I think and feel about other experiences.	1.00	1.00	1.00	1.00
5	This event permanently changed my life.	1.00	1.00	1.00	1.00
6	I often think about the effects this event will have on my future.	1.00	1.00	1.00	1.00
7	This event was a turning point in my life.	1.00	1.00	0.87	0.75

^a^Content validity index;

^b^Content validity ratio.

In this formula, *ne* is the number of experts who selected the score 3 for an item, and *N* is the total number of experts. If the value of CVR for an indicator is equal to or greater than 0.75, then that item has acceptable content validity (Lawshe, 1975). As shown in Table 2, the CVR values for all indicators were above 0.75, indicating an acceptable level of content validity for all items.

### 3.3 Data analysis

Descriptive analyses of the data were conducted using the Statistical Package for the Social Science (SPSS version 25), and preliminary analyses (missing data, normality, and outliers), the Confirmatory Factor Analysis (CFA), the Average Variance Extracted (AVE), the Composite Reliability (CR), and the Correlation Coefficients were conducted using the Asset Management Operating System (AMOS 24).

### 3.4 Preliminary analysis

There were no missing data in the dataset. In addition, outliers were checked using the Mahalanobis D2 value. Dividing the largest D2 value (26.465) by the total number of items (7) resulted in a value of 3.78 (less than 4), indicating that there were no outliers in the data set (Tabachnick & Fidell, 2014). The results of the normality analysis showed that the skewness (−0.90 to −0.21) and kurtosis (−1.23 to 0.45) values were in the range of ±2 and ±3, respectively, confirming the normal distribution of the data (Tabachnick & Fidell, 2014).

### 3.5 Construct validity

**FIGURE 1:**
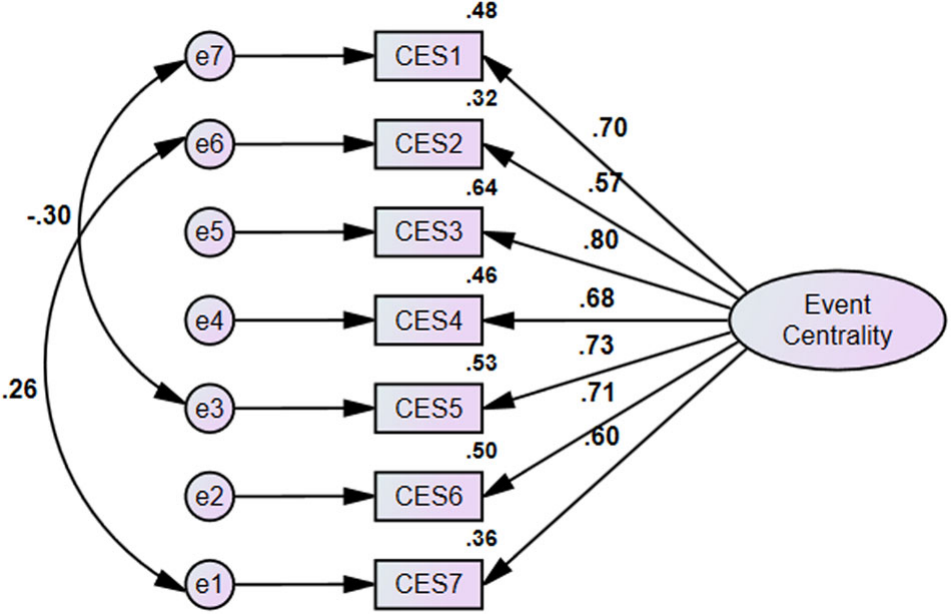
Confirmatory factor analysis with factor loadings for the CES (*p* < .001)

The hypothesized relationships between the items and the CES factor were examined using the CFA in the AMOS software. The assessment of construct validity comprised three parts. In the first part, the factor loadings of the indicators were checked. According to Kline (2015), the factor loading values should not be less than 0.5, greater than 1, or negative. As shown in Figure 1, the factor loading values of all items met these criteria. Therefore, all items remained in the CES. The means and standard deviations of the items are presented in Table 3. In the second part, the following measurement fit indices were checked: CMIN/df < 5; goodness of fit index (GFI) > 0.90; comparative fit index (CFI) > 0.90; Tucker–Lewis Index (TLI) > 0.90, normed fit index (NFI) > 0.90, incremental fit index (IFI) > 0.90, root mean squared error of approximation (RMSEA), and standardized root mean square residual (SRMR) < 0.08 (Bryne, 2010). The results of the measurement model fit indices revealed that the 7‐item CES with a one‐factor structure fit the data adequately (CMIN/df = 4.36, *p* < 0.01, GFI = 0.97, CFI = 0.97, TLI = 0.95, NFI = 0.96, IFI = 0.97, RMSEA = 0.08, SRMR = 0.034). In the third part, convergent validity was calculated by average variance extracted (AVE), which resulted in a value of 0.47. The internal consistency and reliability of the 7‐item CES were examined by the composite reliability (CR) value and Cronbach's alpha coefficient. The values of CR (0.81) and Cronbach's alpha (0.86) were greater than 0.7 (see Table 4), indicating that the 7‐item CES has good convergent validity, internal consistency, and construct reliability (Tabachnick & Fidell, 2014).

**TABLE 3:** Means and standard deviations of the items of centrality of event scale (CES)

No	Items	Mean	Std. deviation
1	I feel that this event has become part of my identity.	3.47	1.22
2	This event has become a reference point for the way I understand myself and the world.	3.56	1.09
3	I feel that this event has become a central part of my life story.	3.70	1.19
4	This event has colored the way I think and feel about other experiences.	3.76	1.03
5	This event permanently changed my life.	3.21	1.37
6	I often think about the effects this event will have on my future.	3.41	1.33
7	This event was a turning point in my life.	3.40	1.20

**TABLE 4:** Correlations between the studied variables

Variables	1	2	3	4	5	6	7
1) Centrality of event	1						
2) Psychological inflexibility	0.45**	1					
3) PTSD	0.54**	0.69**	1				
4) Intrusion	0.48**	0.53**	0.84**	1			
5) Avoidance	0.22**	0.15**	0.46**	0.32**	1		
6) Negative alterations in mood and cognitions	0.49**	0.70**	0.91**	0.65**	0.29**	1	
7) Hyperarousal	0.46**	0.62**	0.88**	0.66**	0.29**	0.72**	1

** Significant at the 0.01 level.

### 3.6 Concurrent validity

The results of the Pearson correlation analysis showed that the 7‐item CES had significant positive relationships with psychological inflexibility, PTSD, intrusion, avoidance, negative alterations in cognition and mood, and hyperarousal (see Table 4).

### 3.7 Measurement invariance

Measurement invariance (MI) refers to the psychometric equality of a construct between groups. Testing measurement invariance illustrates whether indicators assess the same concept across different groups. Since participants in various groups (e.g., gender, cultural, or ethnic groups) may interpret the meaning or function of a construct differently, testing for measurement invariance seems necessary. Testing measurement invariance is a prerequisite for comparing group means, especially in psychological and developmental research (Putnick & Bornstein, 2016). Therefore, it seems essential to test the measurement model invariance of the 7‐item CES before comparing means between gender groups. Because the sample size was unbalanced across gender groups (females = 400, males = 125), 125 females were randomly selected using SPSS before testing measurement invariance and gender differences. Testing for measurement invariance was performed according to the instructions of Bryne (2010). First, *configural invariance* was tested to determine if the factor structure of the measurement model was the same across gender groups. The results of the multigroup analyses in the AMOS software demonstrated acceptable model fit for both gender groups in CES (CMIN = 62.687, df = 26, CMIN/df = 2.41, *p* < .01, GFI = 0.94, CFI = 0.95, RMSEA = 0.075). *Metric invariance* was then tested by constraining the factor loadings in both groups. The results of the chi‐square difference test showed a non‐significant decrease in the X^2^ value compared to the configural model (*p* = .416). Finally, *scalar invariance* was tested by constraining factor loadings and item intercepts in both gender groups. Again, the non‐significant decrease in the X^2^ value compared to the metric model indicated full scalar invariance between the two groups. Once scalar invariance was established, the means of CES scores were compared between the gender groups. The results of the latent mean comparisons showed no significant differences between males and females in event centrality (CR = 0.552; *p *= .581).

## 4 DISCUSSION

The present study aimed to translate the 7‐item version of the CES into Persian and to examine the psychometric properties of this translated version among Iranian university students who had experienced a romantic breakup in the past 2 years. The 7‐item CES was translated from English into Persian using the Brislin method, and the translators confirmed the consistency between the Persian version and the original version.

The results of face validity using the impact score index verified that the indicators of the 7‐item CES were understandable and relevant from the participants’ perspective. The results of the qualitative and quantitative content validity analysis suggested that the translated indicators of the 7‐item CES appropriately assessed event centrality among Iranian university students. In line with the first hypothesis and the theoretical framework for CES (Berntsen & Rubin, 2006), the results of the construct validity confirmed a one‐factor model with high internal consistency and good fit indices in the studied sample. This finding aligns with the previous studies demonstrating satisfactory psychometric properties of the short version of the CES (Berntsen & Rubin, 2006; Galán et al., 2017; Gauer et al., 2013; Vagos et al., 2018; Vermeulen et al., 2020). The factor loading of all indicators were above 0.5 (ranging from 0.57 to 0.80). Consequently, all items remained on the scale. The coefficients of Cronbach's alpha (0.86) and CR (0.81) represented acceptable internal consistency. The acceptable Cronbach's alpha value in the current study is similar to that reported in the measure development study (0.88; Berntsen & Rubin, 2006).

In line with the second hypothesis, higher CES scores correlated positively with PTSD symptoms. To better understand the relationship between event centrality and PTSD symptomatology, correlations between CES scores, total PTSD, and PTSD subscales were investigated. Results showed that all B, C, D, and E symptoms of PTSD were correlated with CES. The positive relationship between CES and PTSD symptoms has been well documented in previous studies (Berntsen & Rubin, 2006, 2007; Boals & Schuettler, 2011; Robinaugh & McNally, 2011; for a review, see Gehrt et al., 2018). The more central a negative event is in the organization of a person's autobiographical memory and personal identity, the more severe the PTSD symptoms the person experiences. This is consistent with Berntsen and Rubin's (2006) model and the cognitive model of PTSD (Ehlers & Clark, 2000), suggesting that the influence of a stressful or traumatic life event on posttraumatic symptoms depends on the individual's perception and appraisal of the event. Another finding is that CES scores are positively associated with psychological inflexibility. This finding is consistent with previous research (Bishop et al., 2018; Boykin et al., 2019; Vagos et al., 2018). Two components of psychological inflexibility are particularly related to the concept of event centrality. Avoidance of event‐related memories, feelings, and thoughts is a non‐functional behavior pattern that reduces the possibility of cognitive processing and resolution of traumatic experiences (Boykin et al., 2019; Vagos et al., 2018). In addition, experiential avoidance results in the use of less effective coping strategies (Chou et al., 2018) and is moderately related to more centralization of an event in one's personal identity (Bishop et al., 2018). Moreover, the self as content process of psychological inflexibility is conceptually similar to event centrality. It refers to the over‐identification with an experience, in which individuals define themselves by their experienced event (Hayes et al., 2012). The traumatized individuals who define their identity as a survivor are at increased risk for developing PTSD symptoms (Boals & Murrell, 2016). Therefore, psychological inflexibility is related to the centralization of an event in one's life and personal identity and increases the risk for developing PTSD symptoms in the face of adversity.

In line with the third hypothesis, the results of the measurement invariance analyses showed that the 7‐item CES is gender invariant and can be used for gender comparisons. This finding is consistent with that of Vagos et al. (2018) who found that the 7‐item CES is gender invariant in adolescents. The current study found configural, metric, and scalar invariances of the 7‐item CES across gender groups in young adults, paving the way for further gender comparisons in CES. Furthermore, the results of the latent mean comparisons revealed no significant gender differences in CES scores. This finding is consistent with recent studies (Berntsen et al., 2011; Cunha et al., 2015; Gauer et al., 2013; Vagos et al., 2018) that reported no significant differences in the centrality of traumatic life events between men and women.

The present study found that the short version of the CES is a valid and reliable instrument for assessing the centrality of traumatic memories in autobiographical memory organization among Iranian university students. This study supports a critical finding of Berntsen and Rubin (2006) that the mechanisms measured by the CES are critical for understanding the importance of traumatic memories in the development of PTSD symptoms. Therefore, this measure may help psychologists and counselors understand the factors involved in the development of posttraumatic symptoms and promote early identification of PTSD following the dissolution of a romantic relationship in university students.

This study also has some limitations. First, a non‐clinical sample of university students was studied. Future research could evaluate the psychometric properties of the 7‐item CES in a clinical sample of individuals diagnosed with PTSD to better understand the role of centrality of negative events in the development and maintenance of PTSD symptoms. Second, this study was conducted on a sample with a specific non‐traumatic life event (i.e., romantic breakup), which limits the generalizability of the findings to other traumatic and stressful events. Future research could seek to validate the use of the 7‐item CES among individuals who have experienced events defined as traumatic in the DSM‐5. Third, this study was restricted to a certain age group (young adults). Future research could consider and compare different age groups in terms of differences in CES. Fourth, given the cross‐sectional nature of this study, causal and temporal conclusions cannot be drawn from the current findings. Longitudinal studies are warranted to examine the directional relationship between event centrality and PTSD symptoms (e.g., is centrality a driving force of PTSD or a response to it?) as well as event centrality and psychological inflexibility (e.g., do inflexible reactions to trauma lead to centralization of an event in one's life or does centrality elicit psychological inflexibility?). In general, more research with different clinical and non‐clinical samples with a history of different negative life events are needed to fully establish the psychometric properties of the 7‐item CES in the Iranian population.

In conclusion, the study revealed that the Persian translation of the 7‐item CES had satisfactory psychometric properties in an Iranian sample of university students with a history of romantic breakup. The results of the CFA support a one‐factor model with good internal consistency. Furthermore, the measurement invariance results confirm that the 7‐item CES is not gender specific and is applicable to both men and women. Therefore, this measure could be used as an effective tool to assess the cognitive impact of significant life events among Iranian university students.

## CONFLICT OF INTEREST

The authors declare no conflict of interest.

### PEER REVIEW

The peer review history for this article is available at https://publons.com/publon/10.1002/brb3.2448

## Data Availability

The data is available at the below link: https://figshare.com/s/1de560deddccf1e0150d
